# Abbreviated lipid guidelines for clinical practice: Based on ESC lipid guidelines 2019 and ESC cardiovascular disease prevention in clinical practice guidelines 2021

**DOI:** 10.1007/s11845-023-03277-x

**Published:** 2023-02-07

**Authors:** Vincent Maher, Joe Gallagher, Ruth Agar, Damian Griffin, Niall Colwell, Patricia O’Connor, Brendan McAdam, Gerald Tomkin, Daphne Owens, Mary Ryan, William Tormey, Maeve Durkan

**Affiliations:** 1 ALMAR Centre, Tallaght University Hospital, Dublin 24, Ireland; 2 Department of General Practice, University College Dublin, Dublin, Ireland; 3 Department of Chemical Pathology, University Hospital Galway, Galway, Ireland; 4 Department of Cardiology, Trinity College Dublin, Dublin, Ireland; 5 Tipperary University Hospital, South Tipperary General Hospital, Clonmel, Ireland; 6 Department of Clinical Pharmacology, St James Hospital, Dublin, Ireland; 7 Beaumont Hospital Dublin, Royal College of Surgeons in Ireland, Dublin, Ireland; 8 Department of Medicine, Trinity College Dublin, Dublin, Ireland; 9 Department of Biochemistry, Royal College of Surgeons in Ireland, Dublin, Ireland; 10 Department of Endocrinology, Bon Secours Hospital, Limerick, Ireland; 11 Department of Chemical Pathology, Beaumont Hospital, Dublin, Ireland; 12 Department of Endocrinology, Bon Secours Hospital, University College Cork, Cork, Ireland

**Keywords:** Abbreviated lipid guidelines, ESC lipid guidelines, Primary care

## Abstract

**Background:**

Lipid disorders are now considered causal for atherosclerotic cardiovascular disease (ASCVD) which remains one of the most important contributors to morbidity and mortality in the developed world. Identification and early treatment of lipid disarrays remains the cornerstone of good clinical practice to prevent, halt and even reverse ASCVD. Guidelines for lipid management are imperative to help promote good clinical practice. Given the detail involved in comprehensive guidelines and the multiple areas of knowledge required by clinical practitioners, abbreviated, easy to understand, practical versions of guidelines are required to ensure dissemination of the most important information. The recent ESC lipid guidelines 2019 and the ESC guidelines on CVD prevention in clinical practice 2021 (1,2), provide an excellent detailed summary of all the latest evidence supporting lipid interventions that reduce ASCVD.

**Method:**

We therefore developed a single-page document with hyperlinks to help practitioners gain easy access to practical information on lipid management. It has been developed for future electronic use in clinical practice.

**Conclusion:**

It is presented here in a tabular format together with printable versions of the associated hyperlinks that provide the additional information required in decision making. It is hoped to audit the impact of this approach to help guide future ways of disseminating the latest clinical guideline updates.

## Introduction

Clinical lipid guidelines based on the latest available information are developed by a panel of international experts [1, 2]. They make recommendations according to the type and level of evidence supporting or not the best course of action in various circumstances. Despite an enormous effort to develop guidelines, their translation into clinical practice is often slow or challenging [3–6]. Many with severe hypercholesterolaemia go undetected or inadequately treated [7]. In the modern era of sound bites and information overload, the practicing physician and nurse have only a limited time to comprehend and utilise guidelines. Previous studies highlight that general practitioners expressed frustration concerning the length and accessibility of guidelines [8]. Despite this, all practitioners hope to have the latest most appropriate information to deliver good clinical practice. In an effort to bridge this gap, many have requested a brief summary, or clinical pathways to help them treat lipid disorders particularly within primary care. The distillation of the extensive knowledge base in guidelines into practical recommendations that are clearly visualised, understood and easy to use requires the collective effort of lipid specialists. Some members of the Irish Lipid Network which incorporates specialists engaged in advanced lipid management in the Republic of Ireland volunteered to undertake this task. They formulated an easy-to-use comprehensive short document with links to appropriate associated information in a manner that could be used electronically in clinical practice. This guide was reviewed and approved by the Quality and Safety in Practice Committee of the Irish College of General Practitioners prior to submission to ensure ease of application in routine general practice.

## Methods

**Fig. 1:**
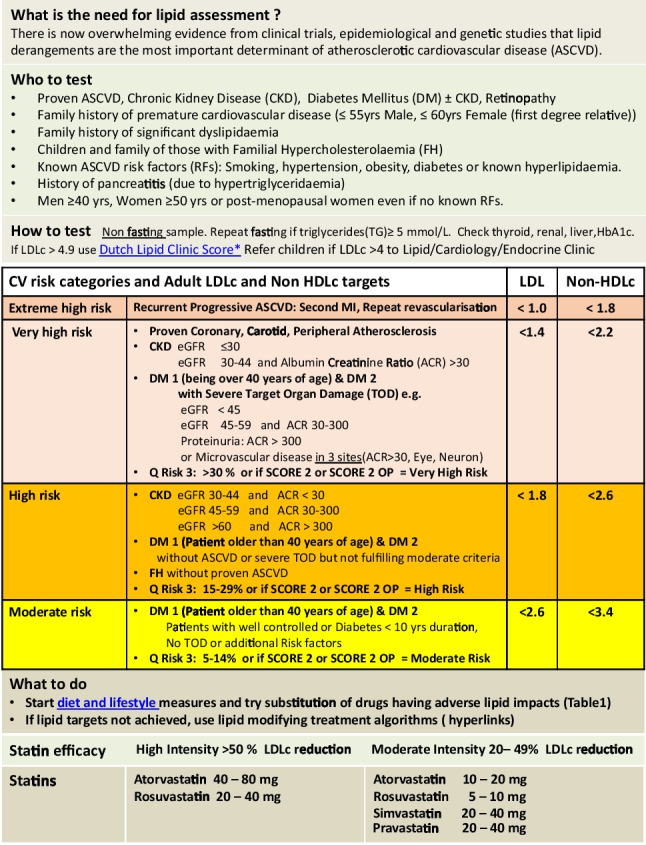
Lipid Guidelines for Adults

**Fig. 2:**
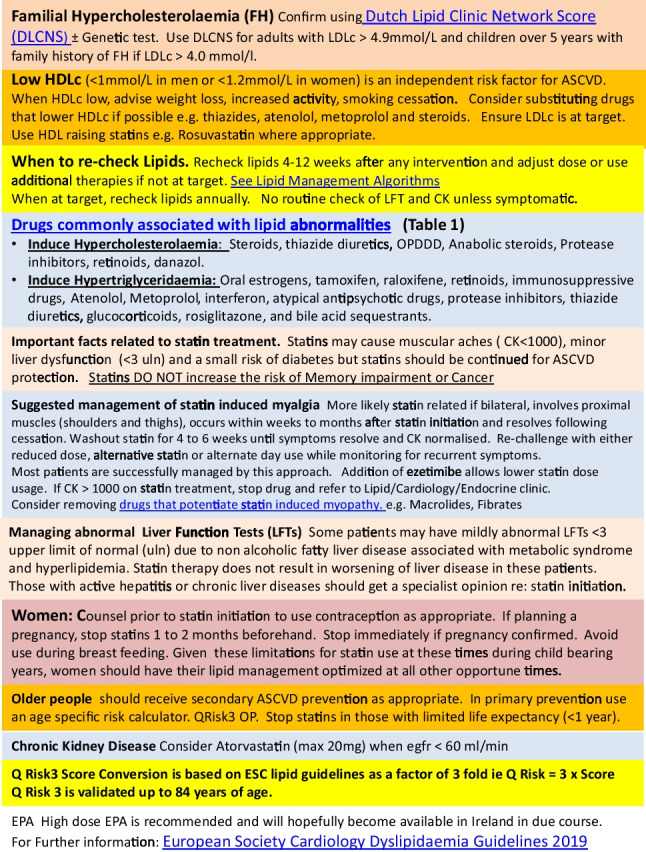
Explanatory notes

A number of members of the Irish Lipid Network were involved in the production of a document that could facilitate interpretation and management of lipid disorders in Ireland. Those involved in specialist lipid clinics are already familiar with the various aspects of lipid guidelines. As a consequence, the focus was on how to produce a short document for the larger body of practitioners particularly those in primary care, to help them manage lipid disorders when time is limited and detailed study is not possible. The specialists who participated included cardiologists, chemical pathologists, endocrinologists, clinical pharmacologists, general practitioners, nurses and scientists. Given the need to widely disseminate lipid management information, general practice was involved from the outset in this process to help guide the appropriate direction undertaken [9]. Following preliminary meetings of a core group of interested parties, a working document was produced. A single paged document (Fig. 1) that could be viewed electronically and easily incorporated into practice computer desk tops was deemed the preferred option. Careful consideration was given to highlight key elements such as the need for abbreviated guidelines, who and how to test, risk groups and target lipid levels, practical steps and suggested lipid-lowering drugs and dosages required to achieve lipid targets. An explanatory page (Fig. 2) was also produced to help guide interpretation when needed. A number of hyperlink materials were also developed to help familiarise practitioners with information that would enhance their understanding of the basis for intervention and need for further screening as deemed necessary [10–15].

### Data tables and hyperlinks

**Fig. 3:**
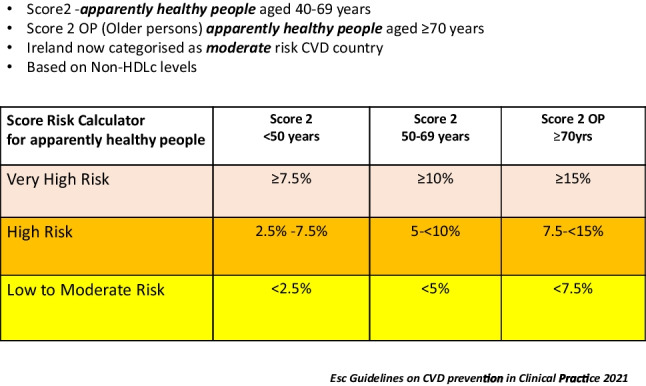
SCORE (Systematic Coronary Risk estimation)

Figure 1 outlines the summarised abbreviated guidelines that will be viewed on a computer desktop. It includes colourised sections to make interpretation easier. Explanatory notes to assist in the interpretation of the abbreviated guidelines are presented in Fig. 2. Risk factors are grouped into moderate, high and very high–risk categories (Fig. 3) using a calculated score from the SCORE 2 and SCORE 2 Older person (OP) charts (2). These charts are used for apparently healthy people living in a moderate CVD risk country (Ireland).

**Fig. 4:**
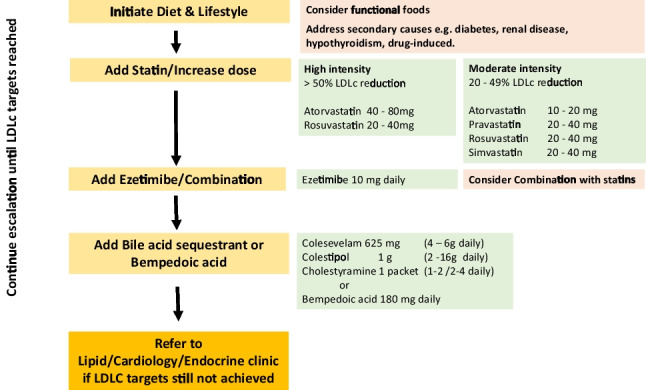
Treatment escalation to achieve LDLc targets

Figure 4 Lipid management algorithm for raised LDL cholesterol levels.

**Fig. 5:**
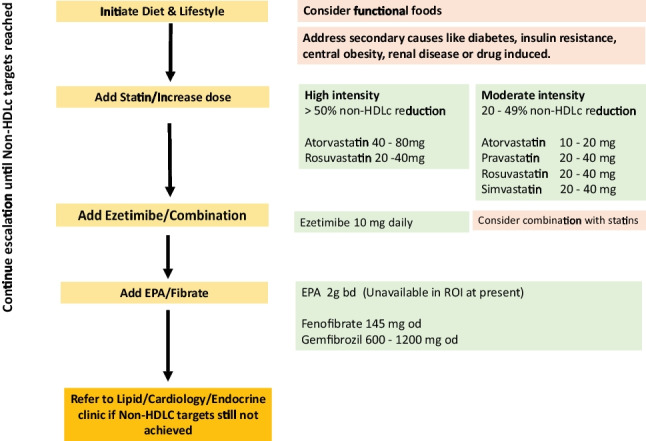
Treatment escalation when triglyceride 2 to 10 mmol/l, HDLc < 1 mmol/l (atherogenic dyslipidaemia; use Non-HDLc targets)

**Fig. 6:**
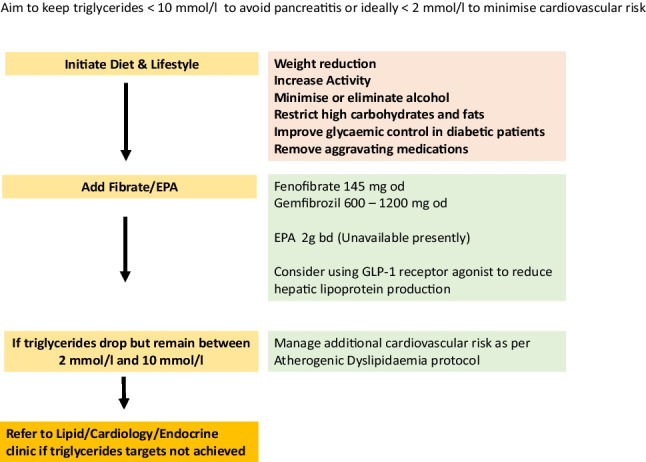
Treatment escalation to achieve triglyceride targets in those with severe hypertriglyceridaemia (TG > 10 mmol/l)

Figure 5 is a flow sheet highlighting the recommended treatment algorithms for those patients with atherogenic dyslipidaemia, and Fig. 6 highlights potential treatment strategies for those with severe hypertriglyceridaemia who are at risk of pancreatitis.

Supplementary information, accessible via hyperlinks from the desktop electronic version of these abbreviated guidelines, is outlined in the appendix as either hyperlink addresses or tables.

**Fig. 7:**
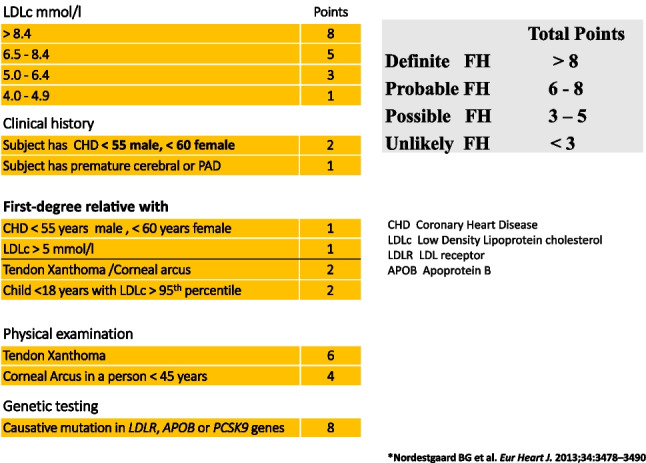
Dutch Lipid Clinic Network Score for familial hypercholesterolaemia (FH)

**Fig. 8:**
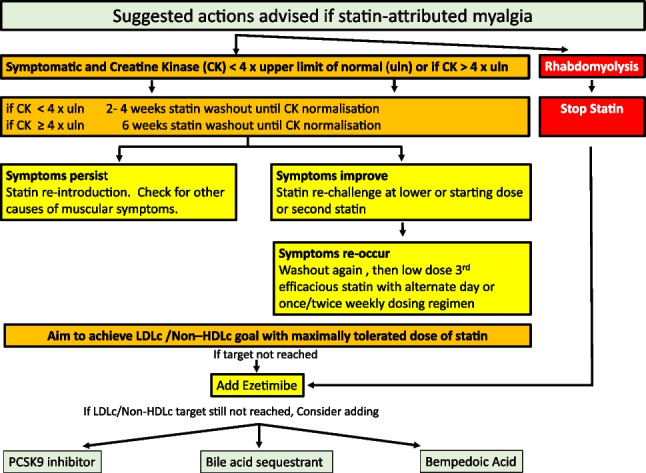
Suggested actions advised if statin-attributed myalgia

These include hyperlink addresses to calculators for Q RISK [10], SCORE 2 and SCORE 2 OP (Ireland moderate risk country) [2] and the Dutch lipid network scoring system (Fig. 7) [11]. The Dutch Lipid Clinic Network score (DCLNS) is a tool intended to support physicians in the diagnosis of heterozygous familial hypercholesterolaemia (FH) in adults. Suggested actions regarding myopathy [12, 13] are revealed in Fig. 8. Drugs that cause hyperlipidaemia [14] are outlined in Table 1. Drugs that interact with statins are outlined in Table 2.

**Table 1:** Drugs commonly associated with lipid abnormalities

**Drugs**	**LDL cholesterol**	**Triglycerides**	**HDL cholesterol**
***Cardiovascular/endocrine***			
Amiodarone	↑Variable	↔	↔
*β*-blockers^c^	↔	↑10–40%	↓5–20%
Loop diuretics	↑5–10%	↑5–10%	↔
Thiazide diuretics (high dose)	↑5–10%	↑5–15%	↔
Sodium-glucose co-transporter 2 (SGLT2) inhibitors	↑3–8%	↔ ↓	↑Variable
***Steroid hormones/anabolic steroids***			
Oestrogen	↓7–20%	↑40%	↑5–20%
Select progestins	↑Variable	↓Variable	↓15–40%
Selective oestrogen receptor modulators	↓10–20%	↑0–30^a^	↔
Danazol	↑10–40%	↔	↓50%
Anabolic steroids	↑20%	↔	↓20–70%
Corticosteroids	↑Variable	↑Variable	↔
***Antiviral therapy***			
Protease inhibitors	↑15–30%	↑15–200%	↔
Direct acting antivirals	↑12–27%	↔	↑14–20%
***Immunosuppressants***			
Cyclosporine and tacrolimus	↑0–50%	↑0–70%	↑0–90%
Corticosteroids	↑Variable	↑Variable	↔
***Centrally acting medications***			
First generation antipsychotics	↔	↑22%	↓20%
**Second generation antipsychotics**	↔	↑20–50%	↔
Anticonvulsants	↑Variable	↔	↑Variable
***Other medications***			
Retinoids	↑15%	↑35–100%	↔ ^b^
Growth hormone	↑10–25%	↔	↔ ↑7%

ESC Dyslipidaemia Guidelines 2019

Atenolol and Metoprolol have adverse effects but not seen with Bisoprolol, Nebivolol or Carvedilol

*LDL* low-density lipoprotein, *HDL* high-density lipoprotein

^a^Raloxifene has not been shown to increase triglyceride levels, while reported increases of up to 30% have been reported with use of tamoxifen

^b^Data remains conflicting and some evidence shows a decrease, no effect, or increase

^c^Varies based on individual drug

**Table 2:** Drugs potentially interacting with statins metabolized by CYP3A4 leading to increased risk of myopathy and rhabdomyolysis

**Anti-infective agents**	**Calcium antagonists**	**Other**
Itraconazole	Verapamil	Cyclosporin
Ketoconazole	Diltiazem	Danazol
Posaconazole	Amlodipine	Amiodarone
Erythromycin		Ranolazine
Clarithromycin		Grapefruit Juice
Telithromycin		Nefazodone
HIV protease inhibitors		Gemfibrozil

2019 ESC/EAS guidelines on the management of dyslipidaemia

The diet and lifestyle are shown in Tables 3 and 4 [1] and are also included in the appendices and contain the latest evidenced-based guidelines from the ESC Dyslipidaemia Guidelines 2019.

**Table 3:** Food choices to lower low density lipoprotein cholesterol and improve overall lipoprotein profile

**To be preferred**		**Use in moderation**	**Use limited amounts**
**Cereals**	Whole grains	Refined bread, rice, and pasta, biscuits, corn flakes	Pastries, muffins, pies, croissants
**Vegetables**	Raw and cooked vegetables	Potatoes	Vegetables prepared in butter or cream
**Legumes**	Lentils, beans, fava beans, peas, chickpeas, soybean		
**Fruit**	Fresh or frozen fruit	Dried fruit, jelly, jam, canned fruit, sorbets, ice lollies/popsicles, fruit juice	
**Sweets and sweeteners**	Non-caloric sweeteners	Sucrose, honey, chocolate, sweets/candies	Cakes, ice creams, fructose, soft drinks
**Meat and fish**	Lean and oily fish, poultry without skin	Lean cuts of beef, lamb, pork, and veal, seafood, shellfish	Sausages, salami, bacon, spare ribs, hot dogs, organ meats
**Dairy food and eggs**	Skimmed milk and yoghurt	Low-fat milk, low-fat cheese and other milk products, eggs	Regular cheese, cream, whole milk and yoghurt
**Cooking fat and dressings**	Vinegar, mustard, fat-free dressings	Olive oil, non-tropical vegetable oils, soft margarines, salad dressing, mayonnaise, ketchup	Trans fats and hard margarines (better to avoid them), palm and coconut oils, butter, lard, bacon fat
**Nuts/seeds**		All, unsalted (except coconut)	Coconut
**Cooking procedures**	Grilling, boiling, steaming	Stir-frying, roasting	Frying

ESC dyslipidaemia guidelines 2019

**Table 4:** Food and lifestyle choices to improve Lipid profiles

**Lifestyle interventions**	**Magnitude of effect**	**Level of evidence**
**To reduce TC and LDL-C levels**		
Avoid dietary trans fats	**++ **	A
Reduce dietary saturated fats	**++ **	A
Increase dietary fibre	**++ **	A
Use functional foods enriched with phytosterols	**++ **	A
Use red yeast rice nutraceuticals (not with statin)	**++ **	A
Reduce excessive body weight	**++ **	A
Reduce dietary cholesterol	**+ **	B
Increase habitual physical activity	**+ **	B
**To reduce triglyceride-rich levels**		
Reduce excessive body weight	**+ **	A
Reduce alcohol intake	**+++ **	A
Increase habitual physical activity	**++ **	A
Reduce total amount of dietary carbohydrates	**++ **	A
Use supplements of n-3 polyunsaturated fats	**++ **	A
Reduce intake of mono- and disaccharides	**++ **	B
Replace saturated fats with mono/polyunsaturated fats	**+ **	B
**To increase HDLc levels**		
Avoid dietary trans fats	**++ **	A
Increase habitual physical activity	**+++ **	A
Reduce excessive body weight	**++ **	A
Reduce carbohydrates: replace with unsaturated fats	**++ **	A
Modest consumption of alcohol may be continued	**++ **	B
Quit smoking	**+ **	B

ESC Dyslipidaemia guidelines 2019. Level A: data derived from multiple randomized clinical trials or meta- analysis. Level B: data derived from a single randomized clinical trial or large non randomized studies

+ + + > > 10%, + + 5–10%, + ≤ 5%

Having electronic versions of these guidelines and associated materials provides a buildable platform which will enable continuous updates as new information and evidence arises.

## Discussion

The extent of lipid problems in Ireland is significant, and lipid management [4, 6] and lipid services are inadequate [15, 16]. The significance of untreated lipid disorders on population health cannot be overestimated [17, 18], and early interventions could have far-reaching consequences on Irish lives and health care resource utilisation. Furthermore, evidence from our older population in Ireland also highlights inadequate treatment of cholesterol problems once diagnosed [19]. Following detection, lowering of LDL cholesterol levels to very low levels yields a 22% cardiovascular risk reduction for each mmol/l LDLc reduction using statins. Additionally, lowering of non-HDLc levels in those with moderately elevated triglycerides further reduces cardiovascular risks [20]. Treatment of those with severe hypertriglyceridaemia markedly reduces the risk of pancreatitis [21].

It is unacceptable that any individual has undetected serious lipid problems which are causing accelerated ASCVD or pancreatitis. As a consequence, when it comes to lipid disorders “What you don’t know may hurt you!”. Therefore, familiarisation of lipid management in primary care will facilitate more individuals being screened and more severe genetic disorders being identified earlier [22, 23]. The objective of these abbreviated guidelines is to facilitate greater understanding by distilling the most pertinent, useable information from the ESC lipid management guidelines (2019) and the ESC Cardiovascular prevention in clinical practice guidelines (2021) related to lipid disorders. Although shortened versions are likely to eliminate a lot of explanatory information that enhances understanding and management, this approach allows interventions to be undertaken quickly in a reassuring manner without the need for further detailed study. For those who wish to glean further information, appropriate links are provided to help in this regard. Since all of this information will be presented electronically on computer desktops, updates and additional information can easily be added. Auditing the use of this abbreviated lipid management tool will facilitate a better understanding of what really matters to practitioners.

As the format of limiting the information to a single page has been widely used in primary care for other conditions, it is likely that this approach to lipid management will also be useful. Endorsement by a group of specialists who are usually the main referral group for lipid management in Ireland may also help utilisation of these modified guidelines. Like all new ventures, only time will tell if this approach is valuable. Certainly, presentation of preliminary versions of these guidelines was widely accepted by general practitioners and endorsed by the Irish College of General Practitioners.

Dissemination of this information and provision of appropriate software versions of these documents for incorporation on computer desk tops will require support. Ideally, monies needed for dissemination of this information should come from various sources to help maintain and expand the development of this tool. Independence from bias on any information provided needs to be ensured in order to engage the trust of practitioners on the validity of this approach long term.

Finally, guidelines are only guidelines, and they will afford practitioners with some of the information required to help their patients. It is likely that these abbreviated guidelines will require associated educational support in order to familiarise practitioners on how to use them and also to gain feedback on their utility. It is envisioned that educational meetings, videos and short practice guidelines will be provided. As a result of increased awareness and interventions, there will also be increasing numbers of patients that need specialist lipid management. In this regard, development of guidelines needs to be followed by the necessary provision of appropriately resourced specialist lipid centres if a seamless optimal lipid management service is to be provided nationally.

## Data Availability

Data is available in referenced guidelines.
